# Melittin Induces Local Order Changes in Artificial and Biological Membranes as Revealed by Spectral Analysis of Laurdan Fluorescence

**DOI:** 10.3390/toxins12110705

**Published:** 2020-11-08

**Authors:** Bogdan Zorilă, George Necula, Mihai Radu, Mihaela Bacalum

**Affiliations:** 1 Department of Life and Environmental Physics, Horia Hulubei National Institute in Physics and Nuclear Engineering, 077125 Măgurele, Romania; bzorila@nipne.ro (B.Z.); mradu@nipne.ro (M.R.); 2 Department of Computational Physics and Information Technologies, Horia Hulubei National Institute in Physics and Nuclear Engineering, 077125 Măgurele, Romania; george.necula@nipne.ro

**Keywords:** melittin, Laurdan, lipid membranes, spectrum deconvolution, molecular simulation

## Abstract

Antimicrobial peptides (AMPs) are a class of molecules widely used in applications on eukaryotic and prokaryotic cells. Independent of the peptide target, all of them need to first pass or interact with the plasma membrane of the cells. In order to have a better image of the peptide action mechanism with respect to the particular features of the membrane it is necessary to better understand the changes induced by AMPs in the membranes. Laurdan, a lipid membrane probe sensitive to polarity changes in the environment, is used in this study for assessing changes induced by melittin, a well-known peptide, both in model and natural lipid membranes. More importantly, we showed that generalized polarization (GP) values are not always efficient or sufficient to properly characterize the changes in the membrane. We proved that a better method to investigate these changes is to use the previously described log-normal deconvolution allowing us to infer other parameters: the difference between the relative areas of elementary peak (ΔS_r_), and the ratio of elementary peaks areas (R_s_). Melittin induced a slight decrease in local membrane fluidity in homogeneous lipid membranes. The addition of cholesterol stabilizes the membrane more in the presence of melittin. An opposite response was observed in the case of heterogeneous lipid membranes in cells, the local order of lipids being diminished. R_S_ proved to be the most sensitive parameter characterizing the local membrane order, allowing us to distinguish among the responses to melittin of both classes of membrane we investigated (liposomes and cellular membranes). Molecular simulation of the melittin pore in homogeneous lipid bilayer suggests that lipids are more closely packed in the proximity of the melittin pore (a smaller area per lipid), supporting the experimental observation.

## 1. Introduction

The increased need to find more efficient candidates as antimicrobial agents has driven researchers to turn their interest towards investigating different classes of molecules. One of them is represented by antimicrobial peptides (AMPs), which display a high efficiency and specificity for various pathogens, especially bacteria [1]. Even though the first AMPs characterized were isolated from a variety of organisms, ranging from prokaryotic organisms to humans, more and more synthetic AMPs have recently been reported [1]. Recently, it was reported that beside antimicrobial activity, some AMPs can also have various activities (anticancer activity, analgesic activity, etc.) and applications in other diseases (autoimmune disease, cardiovascular diseases or treatment of neurodegenerative disease) [2,3,4,5]. In order to perform their therapeutic functions, various methods describing how AMPs interact with the cells were described, ranging from receptor binding peptides, membrane-active peptides to inhibitors of biological pathways [6,7]. Cationic membrane pore-forming AMPs represent a larger subgroup of the AMPs and due to their characteristics are able to kill cells by disrupting their membranes. These peptides are commonly characterized by positive net charge and a hydrophobic core, characteristics which make them membrane active. The first contact of the peptides with the lipid membranes is governed by electrostatic and hydrophobic interactions, conferring them increased affinity for bacterial and cancerous membranes. Following this, based on the structures of peptides, a few models have been proposed to describe peptide-membrane interactions: the barrel-stave model (alamethicin, pardaxin 1), the detergent-like model (LL-37, daptomycin, dermaseptins) and toroidal pores (melittin, magainin) [1,8]. However, their action mechanisms can also be influenced by changes in membrane properties: lipid composition, membrane fluidity or net charge [9]. Based on this, it was reported that one of the resistance mechanism found in bacteria is related to lipid composition modulation [10,11,12].

Considering that plasma membrane has a protective role, for both eukaryotic and bacteria cells, but it also represents the first gateway for peptides to cells, fully understanding the way peptides interact and affect them is essential to help researchers develop more efficient structures in the future.

Melittin (GIGAVLKVLTTGLPALISWIKRKRQQ-NH_2_) is the most studied linear cationic peptide, with a +6 net charge, which adopts an α-helical conformation when inserted into membranes. Besides having a good affinity to bacterial membranes, melittin also shows an increased cytotoxicity against eukaryotic cells and a lytic effect on human red blood cells at concentrations of approximately 2 µM [13,14,15]. Aside from its cytotoxic effect, melittin is known to form channels and induce the deformation or fusion of lipid vesicles [16]. Although in solution melittin adopts a random coil structure, when it binds to the membrane, depending on the experimental conditions, it organizes as a tetramer, which can form either a cylindrical or a toroidal pore [17]. A large number of studies have focused on studying the mechanism underlying the effect of melittin on model lipid membranes [18,19]; however, with only a few papers reporting the effects on the plasma membranes of eukaryotic cells from a biophysical point of view [20].

Due to its good efficiency against bacteria which is equally matched by the lack of specificity for one type of membrane, we were interested to understand melittin effects on lipid bilayers. Thus, we used classical fluorescence spectroscopy to monitor Laurdan membrane probe response to changes in membrane environment. Laurdan is known to show a ~50 nm shift in its emission, from 440 nm to 490 nm, with increased solvent polarity [21]. When incorporated into lipid membranes, previous studies showed that Laurdan can sense membrane polarity changes, thus efficiently detecting local order changes in lipid bilayer, both in model and natural membranes [22,23]. Thus, when the lipid membrane is in the gel phase Laurdan has an emission maximum centered around 440 nm, which shifts to 490 nm when the membrane becomes more fluid. Laurdan generalized polarization (GP) parameters are traditionally used to measure the changes in lipid order of both model and natural membranes by various factors (temperature, drugs, etc. [24,25,26]). Following the recommendation of Jay and Hamilton [27] we refer to this local order as local membrane fluidity.

The aim of the study was to investigate, using Laurdan fluorescence, how small concentrations of melittin affect membrane fluidity in eukaryotic cells by comparison with model membranes, spherical lipid bilayers in the form of large unilamellar vesicles (LUV). In order to better characterize our systems, we used both the classical Laurdan generalized polarization [28] and the new deconvolution method proposed by Bacalum et al. [23]. Considering the complexity of cell membranes, we first studied the effect of increasing concentrations of melittin on different types of liposomes (LUVs prepared from 1,2-dioleoyl-sn-glycero-3-phosphocholine (DOPC) or DOPC/cholesterol and multilamellar lipid vesicles (MLVs) prepared from DOPC/cholesterol). Then we checked the changes induced by melittin in the plasma membrane of four cell lines. The studies were also correlated with the cytotoxic effect of melittin on the same cells. The experimental studies were completed by molecular simulations reporting the lateral packing density of lipid molecules in the absence/presence of melittin.

## 2. Results

### 2.1. Melittin Effect on Cell Viability

**Figure 1:**
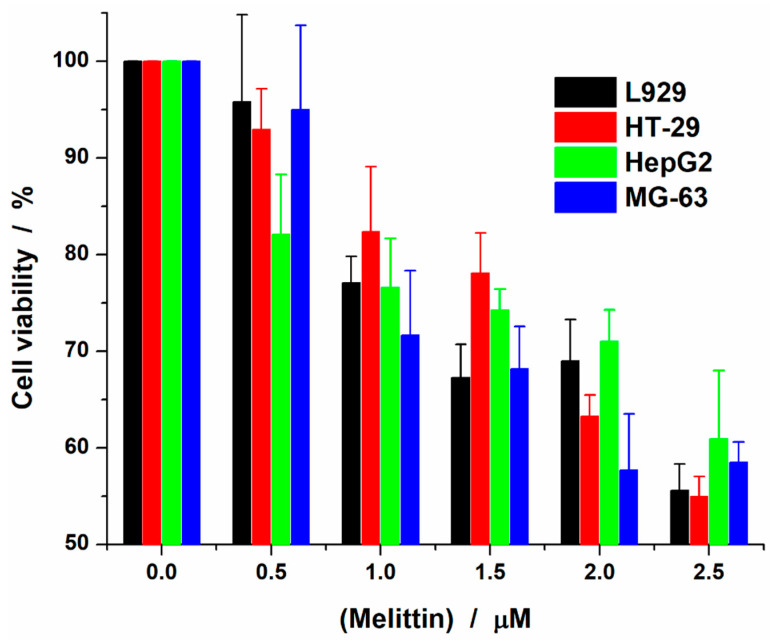
Cell viability after 24 h treatment with varying concentrations of melittin.

First we investigated the effect of melittin on the viability of four eukaryotic cells: L929—mouse fibroblast cells, HT-29—human colon adenocarcinoma, HepG2—human hepatocarcinoma and MG-63—human osteosarcoma (Figure 1). The response of the cells to melittin proved that the peptide has high toxicity towards the cells, at low concentrations, with no significant difference between different cell types. In the investigated range of concentrations (up to 2.5 μM of melittin) melittin did not induce a decrease in viability below 50%. Thus, for the highest concentration tested, cell viability ranged between 55% and 63%. The results suggest, in line with the literature reports that melittin interacts with cells at the lipid fraction of the cell membrane, in a two-step process: binding to the membrane, followed by pore formation [29,30]. Therefore, we further investigated the effect induced by the same melittin concentrations, first on model membranes and finally on cell membranes.

### 2.2. Melittin Effect on Model Membranes

**Figure 2:**
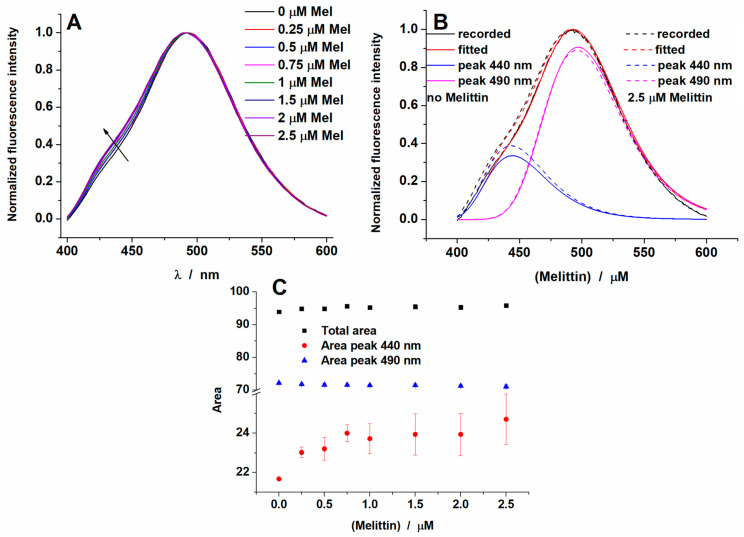
(**A**) Fluorescence emission spectra of Laurdan inserted into 1,2-dioleoyl-sn-glycero-3-phosphocholine (DOPC) liposomes (37 °C) at increasing concentrations of melittin (**B**), deconvoluted spectra for DOPC liposomes alone or in the presence of the highest melittin concentration from (**A**) and (**C**) the dependence of the area values vs. melittin concentration.

Changes in membrane fluidity in DOPC liposomes induced by melittin were evaluated at 37 °C and 15 °C for the same peptide concentration range used in viability experiments. The emission spectrum of Laurdan inserted in DOPC LUVs and recorded at 37 °C is characteristic of Laurdan found in the fluid phase of the membrane, with a prominent emission peak at around 493 nm (Figure 2A). The addition of melittin only slightly affected the emission spectra of Laurdan, the shoulder at 440 nm growing up with increasing melittin concentrations.

Deconvolution in log-normal (LN) elementary peaks of the complex spectra of Laurdan inserted in DOPC liposomes and recorded at 37 °C (Figure 2B) shows a slight increase in the blue peak area, while the green peak area is almost unchanged (Figure 2C). Consequently, the total area follows the blue peak area variation. The blue peak was characterized by a maximum varying between 442 and 444 nm, while the green peak maximum stayed at around 497 nm. The data indicates that addition of melittin results in a higher number of Laurdan molecules being emitted from a more rigid environment. Similar spectral changes were observed for all experimental conditions (data not shown).

**Figure 3:**
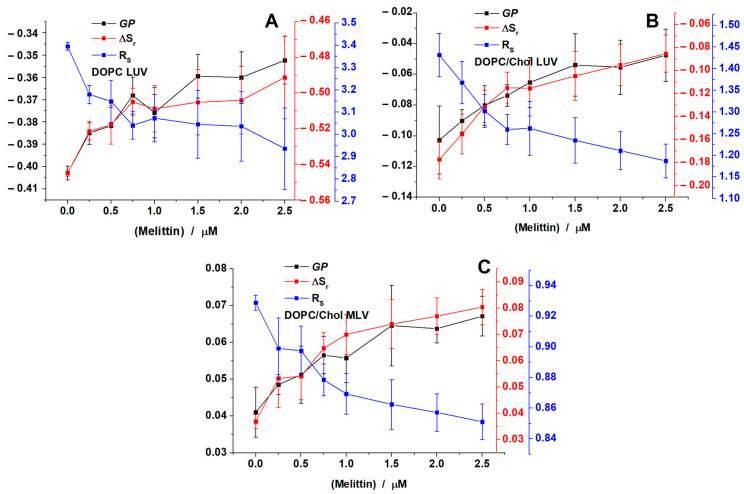
Generalized polarization (GP), ΔS_r_ and R_s_ of Laurdan calculated for DOPC large unilamellar vesicles (LUVs) (**A**), DOPC/Chol LUVs (**B**) and DOPC/Chol multilamellar lipid vesicles (MLVs) (**C**) after the addition of increasing concentrations of melittin and recorded at 37 °C.

Changes have been observed for the parameters calculated by analyzing the emission spectra of Laurdan inserted in the membrane of DOPC liposomes: GP, ΔS_r_ and R_s_ (Figure 3A). The small blue shift seen after peptide addition is noticeable for GP values, which slightly increased from −0.4 for control LUVs to −0.35 for the LUVs found in the presence of 2.5 μM of melittin. A similar increase in GP was obtained for ΔS_r_ as well, ranging from −0.55 to −0.49, respectively. Correspondingly, the addition of melittin induced a decrease in the ratio (R_s_) values from 3.4 for control LUVs to 2.94 for the LUVs treated with the highest concentration of melittin. All these results indicate that the presence of melittin slightly increases the packing of lipid polar heads.

In order to check the effect of cholesterol, lipids normally present in eukaryotic cell membranes, we monitored the effect of melittin on the fluidity of LUVs prepared from DOPC/Chol (Figure 3B). The addition of cholesterol to DOPC membranes induced the increase in membrane organization as expected, with GP values increasing from −0.4 in the case of DOPC LUVs to around −0.1 for DOPC/Chol LUVs. However, the addition of increasing concentrations of peptide inflicted a similar effect on the membrane as in the absence of Chol. Only a small shift to the blue region was observed (data not shown), which was correlated with a slight rigidization of the membrane. Thus, GP values increase from −0.1 for control DOPC/Chol LUVs to −0.05 for the LUVs found in the presence of 2.5 μM of melittin, the GP variation being similar to that found in the absence of Chol. However, the presence of Chol shifted the GP values up, as expected, due to the increasing lipid order induced by Chol (Figure 3A and 3B).

For DOPC/Chol vesicles the LN spectra deconvolution resulted in the blue peak position at around 439 nm, while for the green one, the peak position shifted from 499 nm for control vesicles to 497 nm for those treated with melittin (Figure S2A). For the relative areas, the blue peak area was larger than in the case of DOPC LUVs, due to the presence of cholesterol. Thus, the area increased from 0.41 for control LUVs up to 0.46 after the addition of melittin (Figure S2B). Considering the changes in the area, ΔS_r_ increases from −0.17 to −0.08, while the ratio decreases from 1.43 for control to 1.18 for the highest concentration tested.

To investigate a lipid membrane model more similar to the cells, where internal membranes from organelles are present, we prepared MLVs from a DOPC/Chol mixture (Figure 3C). For this model, GP values increased further to 0.04 as compared to LUVs with similar composition. Similarly to the other two types of vesicles, increasing concentrations of melittin only slightly rigidized the membrane. Thus, GP values increase from 0.04 for control MLVs to 0.06 for the MLVs found in the presence of 2.5 μM of melittin.

For MLVs, the LN spectra deconvolution showed that the blue peak position was found at around 438 nm, while the green one decreased from 500 nm for control MLVs to 498 nm for those treated with melittin (Figure S3A). For the relative areas, the blue peak area was larger than in the case of DOPC/Chol LUVs, being 0.52 for control vesicles and increased with the addition of melittin up to 0.54 (Figure S3B). Considering these values, ΔS_r_ increases from 0.04 to 0.08, respectively, and the ratio decreases only slightly, from 0.93 for control to 0.85 for the last concentration tested.

**Figure 4:**
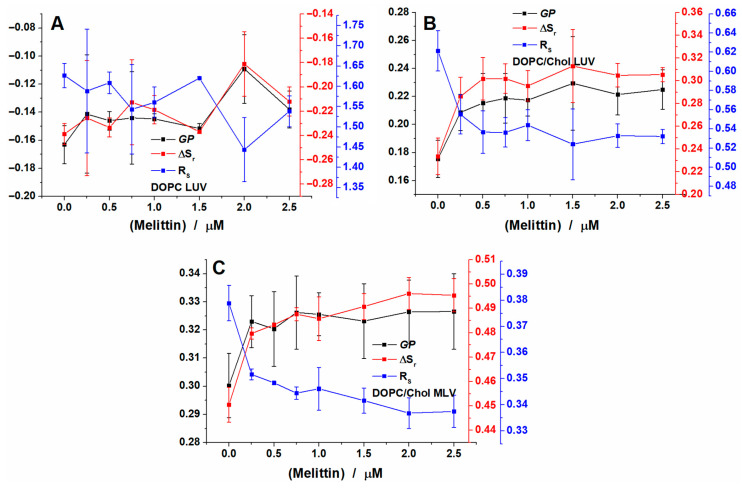
GP, ΔS_r_ and R_s_ of Laurdan calculated for DOPC LUVs (**A**), DOPC/Chol LUVs (**B**) and DOPC/Chol MLVs (**C**) after addition of increasing concentration of melittin and recorded at 15 °C.

Similar recordings were performed at 15 °C. For all lipid systems studied, the decrease in temperature induced an increase in lipid packing. Thus, in Figure 4A we plotted the variation of the three parameters determined for DOPC LUVs. The GP values found for this system at 15 °C are higher compared to those at 37 °C. For control, the value determined is −0.19, while the addition of melittin increases the value. Laurdan peaks are not affected by melittin addition and are centered at 439 nm for the blue one and 495 nm for the green one (Figure S1A). Compared to 37 °C, at 15 °C the area of the molecules found in the blue region increases to 0.39, which is expected as the environment becomes more ordered. Addition of the peptide in this situation increases the population up to 0.4 (Figure S1B).

The GP values plotted in Figure 4B show an increase for control LUVs as compared to those recorded at 37 °C. Thus, the GP values are around 0.18 and increase up to 0.2 after melittin addition. For these vesicles, the blue peak was not affected by melittin addition and was found around 435 nm, while the green one decreased from 497 nm for control LUVs to 494 nm for the ones treated with melittin (Figure S2A). Compared with the same LUVs recorded at 37 °C, at 15 °C, for control LUVs, the area of the blue region increased to 0.62 as compared to 0.41. Melittin addition increased the area even more, up to 0.65 (Figure S2B).

For MLVs, the GP values found at 15 °C are 0.3 for control and increase up to 0.34 after addition of melittin. For MLVs, the blue peak was not affected by melittin addition and was found at around 436 nm, while the green one decreased from 498 nm for control LUVs to 497 nm for those treated with melittin (Figure S3A). Compared with the same vesicles recorded at 37 °C, at 15 °C, for control, the area of the blue region increased to 0.73 as compared to 0.51 and increased further to 0.75 after melittin addition (Figure S3B).

### 2.3. Melittin Effect on Biological Cell Membranes

**Figure 5:**
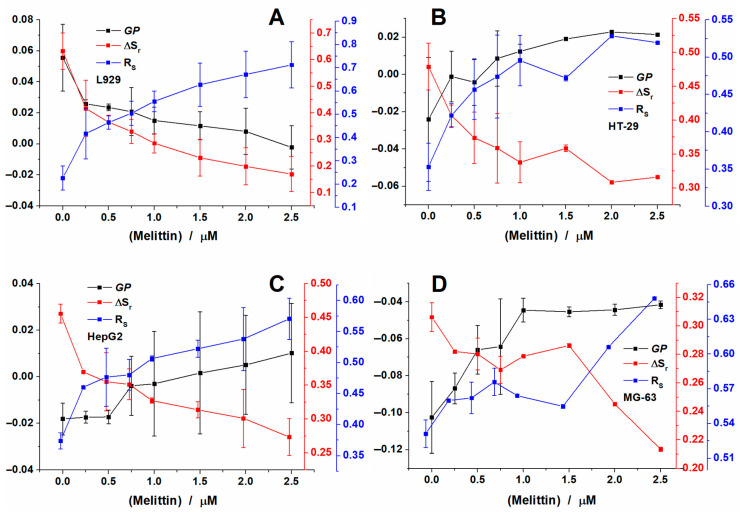
GP, ΔS_r_ and R_S_ of Laurdan calculated for L929 (**A**), HT-29 (**B**), HepG2 (**C**) and MG-63 (**D**) cells after addition of increasing concentrations of melittin and recorded at 37 °C.

Following the study on vesicles, we checked the effect of the peptide on the four cell lines. Depending on the type of cells, therefore a different membrane organization, different effects were observed (Figure 5).

For L929 cells, the addition of melittin induced a decrease in GP values from 0.05 to −0.002 (Figure 5A). The values found for the cells are closer to those found in the case of MLVs. For L929, the addition of melittin induced a similar effect with that observed for all the liposomes recorded.

However, when the spectra were deconvoluted using the two lognormal functions, the two populations obtained were different from the ones obtained for liposomes. The position of the peaks were found for control cells at 456 nm and 511 nm, respectively and melittin addition induced a shift of the two peaks to shorter wavelengths: 446 nm for the blue peak and 500 nm for the green one (Figure S4A). A significant difference was also observed for the behavior of the area of the two peaks. Thus, for the control cells, the area of the blue peak was 0.82 and decreased significantly with the addition of melittin, reaching a value of 0.59 (Figure S4B). This effect is contrary to what we observed for the model vesicles, indicating that cell membrane becomes more fluid after the addition of the peptide. So, when calculating the parameters, contrary to liposomes, ΔS_r_ values decreased from 0.63 to 0.17 with increasing concentrations of melittin, while the ratio increased from 0.22 to 0.71 (Figure 5A).

For the second cell line tested, HT-29, contrary to L929, GP values increased from −0.024 to 0.02 (Figure 5B). Furthermore, the position of the two peaks was found at 451 nm and 506 nm (Figure S5A). After melittin addition the peaks shifted first to longer wavelengths and finally went to the same position. The area of the blue peak was initially of 0.74 and decreased down to 0.66, which is less as compared to L929 (Figure S5B). Thus, ΔS_r_ values decreased only from 0.47 to 0.31 and the ratio values increased from 0.35 to 0.52 (Figure 5B). The results show that the effect was less pronounced in the case of HT-29 cells as compared to L929 cells.

For HepG2 cells, GP values increased from −0.018 to 0.016 (Figure 5C) similarly to the effects observed for HT-29 cells. For control cells, the position of the two peaks was found at 459 nm and 518 nm (Figure S6A) and blue shifted with increasing concentrations of melittin until they reached 451 nm and 508 nm, respectively. Similarly to HT-29 cells, the area of the blue peak is 0.73 and decreased to 0.64 after addition of melittin (Figure S6B) and so, the ΔSr values decreased from 0.46 to 0.27 and the ratio values increased from 0.37 to 0.57. The results support the image that melittin affects both HT-29 and HepG2 cells in a similar manner.

In the case of MG-63 cells, the GP values found were the lowest compared with the other three cell lines, however, the values increased from −0.10 to −0.041 with melittin addition (Figure 5D). The two peaks found were characterized by maxima found at 462 nm and 520 nm and were affected by melittin reaching 454 nm and 511 nm (Figure S7A). The area of the blue peak was 0.65 for control cells, and decreased to 0.61 for the last condition tested (Figure S7B). Thus, the ΔS_r_ values decreased from 0.31 to 0.21 and the ratio values increased from 0.53 to 0.64. The results indicate that the peptide had the smallest effect on this cell line.

### 2.4. Area Per Lipid and Membrane Hydration Analysis

The lateral packing density of lipid molecules is an important parameter that has a significant influence on the structural characteristics of membranes. Lateral packing density was assessed by calculating the average DOPC area per lipid of the two systems (melittin embedded in DOPC and pure DOPC membrane).

In order to assess the convergence and fluctuations of the membrane during the simulations, a general assessment of APL was performed on the entire trajectory, in 10 ns intervals, revealing an average APL of 0.63 ± 0.016 nm^2^ for the melittin system and 0.683 ± 0.012 nm^2^ for the pure DOPC systems. While the pure DOPC membrane achieved convergence after only 40 ns, the membrane with the embedded melittin channel, achieved convergence much later, at 400 ns.

**Figure 6:**
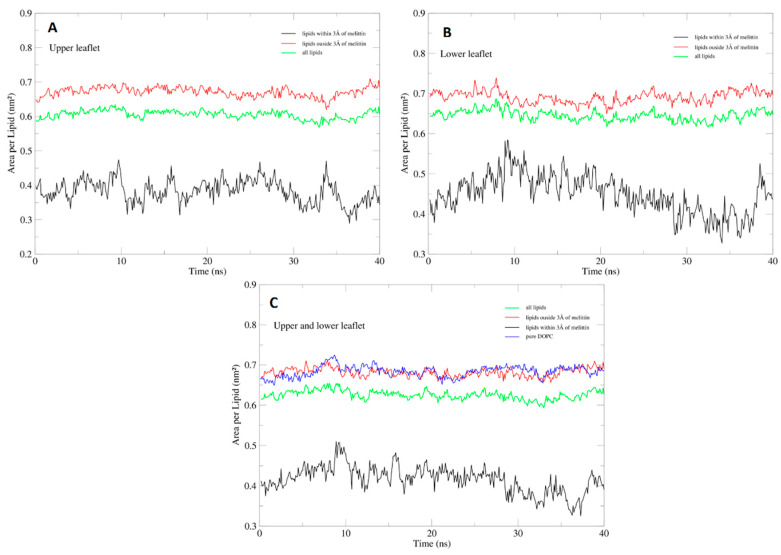
Average APL for the two regions of DOPC membrane containing the melittin channel in the upper leaflet (**A**), lower leaflet (**B**) and upper and lower leaflet (**C**) with comparison to the pure DOPC membrane.

To evaluate the extent to which the presence of the melittin channel affected the lateral packing density of the membrane during the simulation, we performed an APL analysis on the both leaflets of the membranes. DOPC molecules within an area of 3 Å around the center of mass of the melittin channel (approximately 20 average lipids per leaflet), exhibited a considerable reduction of average APL to 0.384 ± 0.032 nm^2^ for the upper leaflet (Figure 6A) and 0.448 ± 0.048 nm^2^ for the lower leaflet (Figure 6B), with average APL on both leaflets of 0.417 ± 0.032 nm^2^. The lateral packing density of lipids outside the 3 Å selection was largely unaffected by the presence of melittin: APL of 0.671 ± 0.013 nm^2^ for the upper leaflet and 0.691 ± 0.014 nm^2^ for the lower leaflet, while the average APL for both leaflets was at 0.681 ± 0.01 nm^2^. The average APL for both leaflets of the pure DOPC membrane was very similar 0.683 ± 0.013 nm^2^ (Figure 6C). The overall average APL was reduced as a direct consequence of the presence of melittin to 0.605 ± 0.012 nm^2^ for the upper leaflet, 0.645 ± 0.013 nm^2^ for the lower leaflet and 0.625 ± 0.011 nm^2^ for both leaflets (Figure 6C).

**Figure 7:**
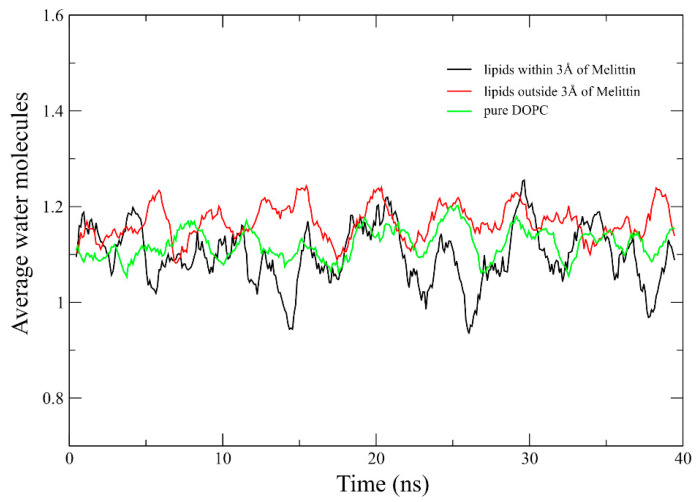
Average water insertion at the maximum observed depth into the two regions of membrane containing the melittin channel, respectively in pure DOPC membrane.

To confirm the APL derived lipid packing density results, we also performed analysis of water penetration into the membrane models. We observed a reduced propensity of water molecules to penetrate the lipids in close proximity (identical 3 Å DOPC selection) to the melittin channel. This finding correlates with the reduction of APL in this area, in other words, an increased lateral packing of lipids that hinders water penetration to the maximum observed depth revealed in Molecular Dynamics (MD) simulation. The average water insertions into the membrane area in close proximity to the melittin channel was 1.098 ± 0.134 water molecules and 1.165 ± 0.074 for the rest of the membrane, respectively 1.122 ± 0.063 in pure DOPC membrane. Although there was a limited reduction of average water insertions into the membrane near the melittin channel, in the 40 ns time frame, there were instances of up to 2.25 ns with a reduction by as much as 28.7% of membrane hydration level near the hydrophobic region of the membrane (Figure 7).

## 3. Discussion

Antimicrobial peptide-lipid membrane interactions are responsible for various processes, which can be strictly localized at the plasma membrane level (membrane disruption, membrane fusion, etc.) or influence the membrane-mediated cellular processes (membrane translocation, interaction with membrane receptors, etc.) [31,32]. These processes are controlled on one side by membrane organization and on the other by peptides structure and properties. Considering the complexity of the processes mediated by peptide-membrane interactions, studies involving this topic are challenging, but even so, the information could help unravel the mechanisms involving peptides.

To date, there is a substantial amount of studies reporting antimicrobial peptides interaction with lipid systems [25,32,33,34,35,36,37,38,39]. However, there is only a small number of biophysical studies performed in vitro, mostly on bacterial cells [40,41,42,43], and only a few on eukaryotic cells [20].

For these biophysical studies we were interested in the effect induced by small concentrations of peptides into the membranes of lipid vesicles and cell membranes. Based on the in vitro studies we initially performed concerning the viability of cell cultures treated with melittin, we chose the concentrations which did not dramatically affect the viability, being applied sufficiently for the peptide to insert into the membrane (survival rate over ~60%). Melittin was applied up to 2.5 µM for both type of membranes.

To test the changes in local order of lipid bilayer we used the very common method of fluorescence spectroscopy based on Laurdan membrane probe. Indeed, there are other more sophisticated biophysical techniques to record the response of the Laurdan molecule embedded into the local environment of the lipid bilayer (methods based on fluorescence microscopy or fluorescence lifetime spectroscopy [44,45]), but here we chose to follow the most popular way to address the Laurdan response. Melittin addition to model membranes induces a small shift to the Laurdan spectra. A blue shift was previously reported for PC and OE7 membranes when melittin, mastoparan and alamethicin are added [46]. However, Dinic et al. [25] reported that Laurdan small blue shifts are not caused by peptide presence (mastoparan and bPrPp). Contrary to their report, our results show that although small, the changes in the emission spectra of Laurdan is induced by the melittin insertion into the membrane. Classical GP was calculated, as well as the ΔS_r_ and R_S_ parameters obtained after LN deconvolution as previously described [23]. For all experimental conditions investigated on model membranes (LUVs and MLVs), melittin addition enhanced the lipid membrane order locally regardless of the type of liposomes or the temperature. Both the GP and ΔS_r_ increased with increasing melittin concentrations, while R_S_ values decreased.

When melittin was added to cells, GP values increased with increasing concentrations, suggesting a stiffening of the plasma membrane similar to what we observed for liposomes. One exception is for L929 fibroblast cells, which were characterized by a fluidization of the plasma membrane with melittin addition. The difference in the results has to be determined by the difference in membrane composition, for both vesicles and cells. For liver and colon cells, studies reported that the amount of cholesterol from the total lipids is varying between 17% and 21%, while phospholipids are between 55% and 62%, while for fibroblasts the cholesterol/lipid ratio is almost double [47]. Based on the results we could say that MG-63 cells have a lipid membrane composition which makes them more fluid, compared with the other cells. HT-29 and HepG2 have a similar composition, thus we also found a similar trend for GP values, while L929 had the most rigid membrane. Considering their roles in the organism and lipid membrane composition, these findings were somewhat expected.

**Figure 8:**
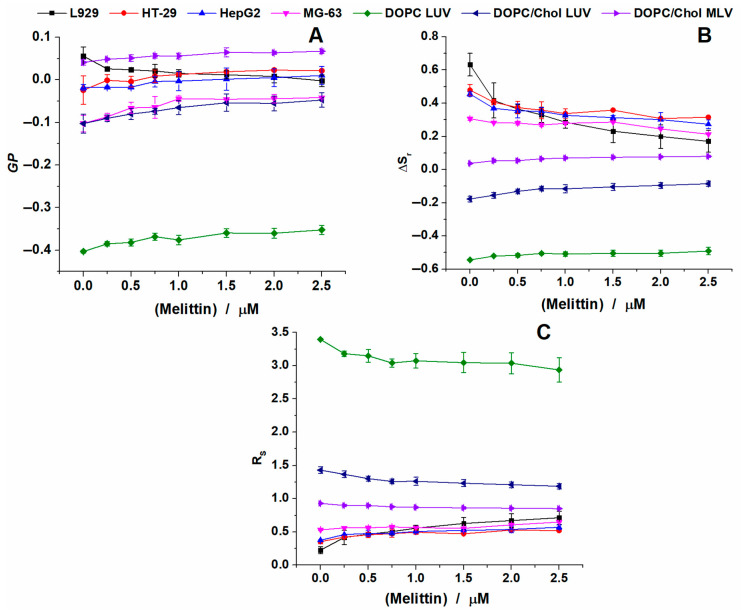
Effect of increasing concentrations of melittin on the GP (**A**), ΔS_r_ (**B**) and R_S_ (**C**) of Laurdan embedded in different lipid vesicles and cells at 37 °C.

If we further analyze the results obtained for all experimental conditions without melittin (Figure 8A), we can observe that GP values found for DOPC LUVs are significantly different compared to those reported for all the other conditions, however, it was harder to differentiate between the other vesicles and the cells. MG-63 cells and DOPC/Chol LUVs presented a similar trend, while DOPC/Chol LUVs had the highest GP values reported of the tested conditions. Based on the complexity of the plasma membrane of the cells, we would expect to have a better differentiation between the model membranes and the natural ones.

Surprisingly, ΔS_r_ and R_S_ had an opposite trend as compared with that reported for the vesicles (Figure 8B,C). This is caused by the different trend observed for the two peaks obtained after deconvolution. For vesicles, the area of the blue peak increased with increasing concentrations of melittin, while for the cells, the area of the blue peak decreased with melittin addition. However, when we analyze the results (Figure 8B,C) one can see that these parameters (ΔS_r_ and R_S_) allow us to differentiate better between vesicles and cell membranes. We have clearly differentiated the three different vesicles’ responses, even after melittin addition, with respect to the plasma membrane of the four cell types studied.

**Figure 9:**
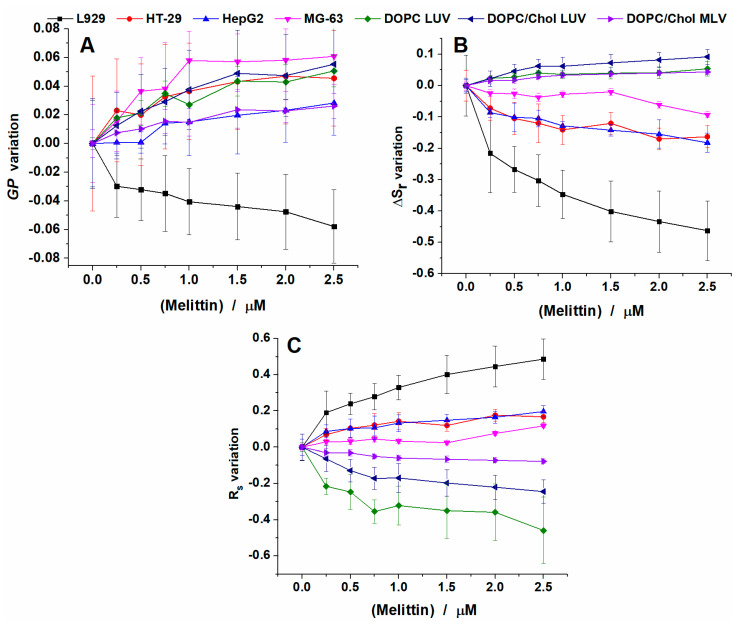
Differences between GP (**A**), ΔS_r_ (**B**) and R_S_ (**C**) values with increasing concentrations of melittin in lipid vesicles and cells at 37 °C.

To better compare the changes observed for all parameters calculated, we plotted the absolute variation of GP, ΔS_r_ and R_S_ values with increasing concentrations of melittin (Figure 9). Similar discussions were reported previously for GP [35,48].

For GP values, the most surprising effect was observed for L929 cells. Although for the other three cell lines, the addition of the peptide induced an increase in GP values, thus an increase in the packing of the lipids; in the case of L929 cells, the addition of melittin induced a decrease in GP values, thus a fluidization of the membrane.

When checking the changes in GP (Figure 9A), for all conditions tested, except for L929 cells, almost no differences and a similar trend are shown: compared with the control condition (without melittin), the addition of melittin will induce an increase in the GP values, thus a stiffening of the membranes in a similar trend with no distinction between different types of vesicles and cells.

However, if we check the ΔS_r_ and R_S_ variations (Figure 9B,C) we can see a different trend between model membranes and biological membranes.

For ΔS_r_ we see that the highest change is for L929, which produced a decrease of 0.5 compared to the control condition. For the other three cell lines a similar effect was observed, but the changes were less pronounced: around 0.1 for HT29 and HepG2 cells and below 0.1 for MG-63 cells. On the contrary, for liposomes, ΔS_r_ increased with amounts between 0.05 and 0.1.

This difference in cell membrane behavior with respect to model membranes after melittin treatment is also revealed by R_S_. For DOPC LUVs, melittin addition induces a decrease in R_S_ by 0.5 at the highest concentration tested. The effects are less for DOPC/Chol LUVs, with a decrease of 0.2 and even smaller for DOPC/Chol MLVs, around 0.05. The differences in the effect are correlated with the changes in membrane complexity and rigidity. When we look at the cells, we find that melittin addition increases the values of R_S_, by 0.1 up to 0.2 for MG-63, HT-29 and HepG2 cells, while for L929 up to 0.5. The reasons for this opposite response of natural membranes compared with the liposome one could be found in the heterogeneity of the lipid bilayers in cellular membranes, particularly the presence of lipid microdomains (rafts). McHenry and colleagues proved that the presence of cholesterol in a binary lipid bilayer where the separation of phases occurs did not protect the membrane against the action of pore-forming AMPs. However, the presence of cholesterol reduced AMP’s effect in the homogeneous membrane [49]. Similarly, the presence of cholesterol in the homogenous phase of DOPC LUVs supports the stability of the membrane in the presence of melittin in our experiments, while an opposite effect is observed in the heterogeneous lipid bilayer of cells. We may speculate that the lack of protective effect of cholesterol in the cell membrane leads to an enhanced local disorder in the lipid bilayer as our results suggest (Figure 9B,C). This explanation is indirectly supported by the observation that all the model membranes have smaller values of ΔS_r_ (or higher values of R_S_ according to Figure 8B,C) proving that homogeneous model membranes are more fluid than the heterogenous lipid bilayer in natural membranes where the lipid rafts is presumed to exist. These variations are indicative of a different environment in the membrane after melittin addition. Using R_S_ variation, we can easily differentiate between the different membranes as well as the increasing treatment concentration.

In silico methods were used to investigate the area per lipid and the membrane hydration in presence of melittin. A considerable reduction of lateral packing density of lipids in close proximity of the melittin channel embedded in DOPC model membrane was observed, indicated by the reduction of average area per lipid by 0.26 nm^2^ (from 0.681 to 0.417 nm^2^), and an overall reduction of APL by 0.056 nm^2^ (from 0.681 to 0.625 nm^2^), results confirmed by the analysis of water penetration into the membrane. These findings are in line with literature reports [50,51,52] of antimicrobial peptides reduction of membrane APL as a function of position and structure, and together with the analysis of water penetration into the membrane, support the Laurdan spectroscopy results we report here, the increased lateral packing of lipid polar heads being reflected in the restricted dynamics sampled by Laurdan in the presence of melittin.

A special discussion could be focused on the shape of the Laurdan spectra derived parameters curves vs. melittin concentration. As can easily be observed in Figure 9, the relative change of all parameters (GP, ΔS_r_ and R_S_) shows a monotonical and nonlinear dependence against melittin concentration. A rapid change is present at lower melittin concentrations (under 0.25–0.5 μM) while at higher concentrations the rate of change diminishes. Compared with the viability curve (Figure 1), these small concentrations correspond to very small modification of survival fraction (~90% viability). The main observation here is the fact that this pattern is present in the curves from Figure 9 regardless of model or natural membranes suggesting that melittin rapidly penetrates the lipid bilayer in natural membranes with direct effects on cell viability, the increase of melittin concentration only amplifying this effect in a nonlinear manner.

## 4. Conclusions

The Laurdan parameters (GP, ΔS_r_ and R_S_) reported in the study are able to sense membrane changes after melittin addition. However, when comparing their variation, GP has the smallest one, which did not prove to be sensitive enough sensitive to provide a significant separation between the changes induced by melittin in the different membranes studied. On the other hand, both ΔS_r_ and R_S_ parameters have a larger range of variation and can also show a well-defined distinction between different conditions; R_S_ variation proves to be the most sensitive, both to membrane composition and melittin addition. The molecular simulation proved that lateral packing increases in the presence of melittin in the proximity of the melittin channel simultaneously rendering the water molecules penetration in these regions of the lipid bilayer. The similar changes of Laurdan parameters in model and natural membranes, especially at very small concentrations suggests the localization of the melittin effect at the lipid bilayer level, the difference between the natural and model membranes responses being done by the heterogeneity of the natural ones (the presence of lipid rafts). The approach we presented here can be extended in the future to peptides known to have different action mechanisms.

## 5. Materials and Methods

### 5.1. Materials

Melittin was purchased from Bachem AG (Budendorf, Switzerland). All solvents, DOPC, cholesterol and Laurdan were purchased from Sigma-Aldrich (St. Louis, MO, USA). All culture media were purchased from Biochrom AG (Berlin, Germany).

### 5.2. Cell Viability Assay

Cells were seeded in a 96 well plate at desired densities (8000 cells/well for L929 and MG-63 cells and 15,000 cells/well for HT-29 and HepG2 cells) and grown in medium for 24 h before treatment with different concentrations of melittin ranging between 0 and 2.5 μM in a CO_2_ incubator (37 °C). The following day, cell viability was evaluated using the MTT assay following the protocol described earlier [13]. The cell viability was calculated as a percent value relative to the untreated cells.

### 5.3. Liposomes Preparation and Cell Preparation

DOPC and DOPC/Chol (65:35) LUVs with a final lipid concentration of 50 µM were prepared using the extrusion method as described previously [53]. An appropriate amount of lipids was dried under nitrogen flow to remove the solvent and to obtain a lipid film. The lipid film was hydrated with PBS, heated above the transition temperature (*T_m_*) of the lipids and vigorously vortexed to form a suspension of multilamellar vesicles (MLVs). The MLV suspension was repeatedly frozen–thawed (5 cycles) and extruded (25 times) through a 200-µm filter using a standard extruder (Avanti Polar Lipids, Alabaster, AL, USA). The extrusion was performed at a temperature above the *T_m_* of the lipids, resulting in a suspension of LUVs.

For cell recordings the following protocol was used. The cells were grown in Petri dishes until they reached sub-confluence. Then, the cells were detached using trypsin, centrifuged to remove any trace of medium and trypsin and resuspended in PBS. The cells were kept at 37 °C for the entire duration of the experiment and the experiment did not last longer than 1 h to ensure cells were not killed. At the end of the experiment the cells were observed under an inverted microscope to check if they were still alive.

### 5.4. Laurdan Measurements and Spectra Processing

Steady-state fluorescence measurements were performed using a FluoroMax 3 spectrofluorimeter (Horiba Jobin Yvon, Edison, NJ, USA) equipped with a Peltier thermostated cell holder. The spectra were recorded in the range 400–600 nm, with the excitation set at 378 nm. The slits of excitation and emission monochromators were 3 nm. The recorded spectra were corrected for the spectral sensitivity of the emission channel of the spectrofluorimeter and for Raman and scattering artifacts. Laurdan was added at 100 nM into the LUV and MLV suspensions and 1 μM in cell suspension and left for 20 min at 37 °C to allow full insertion into lipid membranes.

Fluorescence spectra were recorded for every concentration, by adding small aliquots of melittin over liposome solution, leaving them for 10 min, prior spectra measurement, for reaching equilibrium in solution. Data obtained were processed using the OriginPro 2016 software package (OriginLab Corporation, Northampton, MA, USA) to calculate the GP (generalized polarization) parameter as follow:

$$GP=\left(I_{440}-I_{490}\right)/\left(I_{440}+I_{490}\right)$$

where *I*_440_ and *I*_490_ are Laurdan emission intensities at 440 nm and 490 nm.

We used the deconvolution method previously reported [23] to fit the emission spectra of Laurdan with two log-normal (LN) functions: one characterizing the emission of Laurdan molecules found in a more rigid environment (the blue part of the spectra) and the other one the emission of molecules found in the fluid phase (the green part of the spectra). Few parameters have been calculated using the data resulted from spectrum deconvolution: (i) elementary peak positions, (ii) relative areas of the elementary peaks against the total area of the spectrum, (iii) difference between relative areas of elementary peaks (ΔS_r_) and (iv) ratio of elementary peaks areas (R_S_).

### 5.5. Molecular Dynamics Simulation and Analysis

We performed two all-atom molecular dynamics simulations with transmembrane melittin in pure 1,2-dioleoyl-sn-glycero-3-phosphocholine (DOPC) bilayer. For reference, a third, shorter simulation with pure DOPC and without any peptide present was also performed. The initial structure of melittin was downloaded from PCSB Protein Data Bank (PDB ID 6O4M). After a visual inspection of melittin structure, six copies of the peptide were bundled in parallel (N terminals of peptides facing each other) and antiparallel (C and N terminals of adjacent peptides close to each other) orientations to form a hexamer transmembrane channel. The channels with the two orientations of melittin were thoroughly simulated, but only the simulation with the most stable transmembrane channel was analyzed further. All results presented herein are derived from the simulation with melittin arranged in antiparallel orientation, along with the short simulation of pure DOPC membrane.

All three simulations were performed, with minor variations, using the protocol presented below. Each molecular dynamics simulation was performed with NAMD ver. 2.12 [54], using CHARMM36 [55] force fields and TIP3P water model. CHARMM-GUI [56] was used to embed both melittin channels in 180 DOPC lipids (90 per leaflet). The system size along the Z-axis was determined by specifying the thickness of bulk water of 22.5 Å from the protein extent along this coordinate. Counterions were added to neutralize the system and the final ionic concentration was set to 0.15 M NaCl.

After the final assembly of the system, an equilibration protocol was performed to relax the uncorrelated initial system prior to molecular dynamics production simulations: A variety of harmonic restraints were applied to the protein, water, ions and lipid molecules. The harmonic restraints were gradually reduced during the equilibration protocol and were completely removed in the production simulation.

All unrestrained isothermal-isobaric (NPT) production simulations were performed with the following NAMD protocol: Langevin dynamics was used to maintain a constant temperature (310.15 K) and a Nosé-Hoover Langevin-piston was used to maintain constant pressure (1 bar). Periodic boundary conditions were applied and the particle mesh Ewald (PME) algorithm was employed to take into account long range electrostatic interactions. The Van der Waals interactions were smoothly switched off at 10–12 Å. Hydrogen bond lengths were fixed at an ideal value with the SHAKE algorithm and a 2 fs time step was set.

The production runs of 1 µs MD simulations for the two systems with melittin channel and the 200 ns simulation for the system with pure DOPC membrane were performed on two NVIDIA P100 GPUs. Average membrane lipid properties, such as area per lipid (APL) and the analysis of water penetration into the membrane were calculated from snapshots corresponding to the last 40 ns of trajectories, in 100 ps intervals.

Area per lipid (APL) was determined by calculating the partial area of each lipid phosphate head group using GRIDMAT-MD [57], which employs a grid-based tessellation scheme. For each snapshot analyzed, the lipid heads were mapped to a bi-dimensional grid based on the lateral distance of the lipids. Each grid element gets assigned the nearest lipid. APL analysis was executed with 100 × 100 grids, resulting in a grid point resolution of <3 Å^2^.

The penetration of water inside of the DOPC membrane was assessed in Visual Molecular Dynamics (VMD) [58] with a simple Tcl/Tk script that counted water molecules within a distance of 3 Å around the center of mass of atoms O21, C22, O31 and C31 from each DOPC molecule. The analysis MD trajectories revealed that the maximum water insertion depth into the membrane was within a distance of 3 Å to these four atoms of DOPC.

## Supplementary Materials

The following are available online at https://www.mdpi.com/2072-6651/12/11/705/s1, Figure S1–S7: Variation of peak position and area of the peak for liposomes and cells in the presence of increasing concentrations of Melittin.
