# Supplementary material for: Melittin Induces Local Order Changes in Artificial and Biological Membranes as Revealed by Spectral Analysis of Laurdan Fluorescence
Source: Toxins (Basel). 2020 Nov 8;12(11):705. doi: 10.3390/toxins12110705 (PMC7695215; doi:10.3390/toxins12110705)
Supplement: Supplementary file 1 [file toxins-12-00705-s001.pdf]

Supplementary Material: Melittin Induces Local Order Changes in Artificial and Biological Membranes as Revealed by Spectral Analysis of Laurdan Fluorescence

Bogdan Zorila, George Necula, Mihai Radu and Mihaela Bacalum

Laurdan senses Melittin induced fluidity changes in artificial and biological membranes

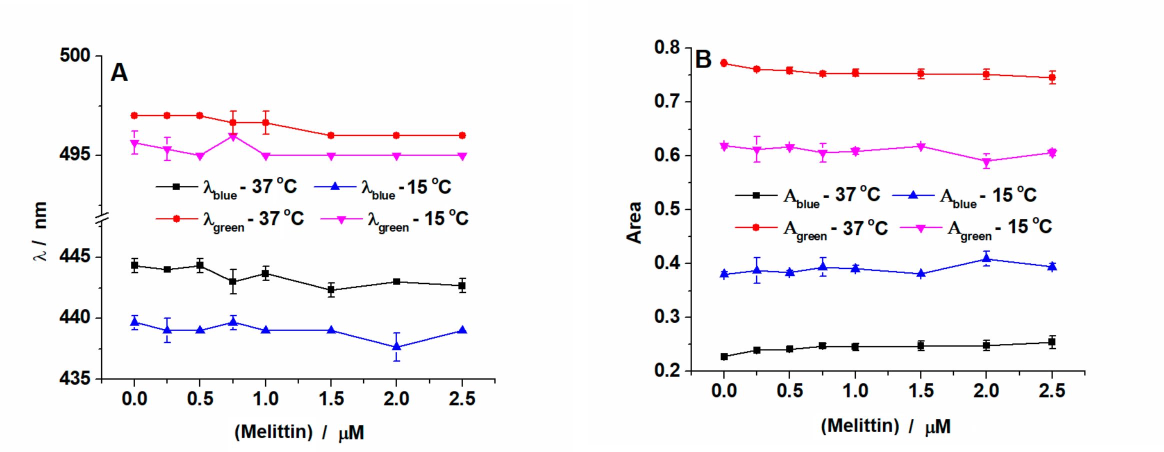

Figure S1. Variation of peak position (**A**) and area of the peak (**B**) for DOPC LUVs recorded at 15 °C and 37 °C in the presence of increasing concentrations of Melittin.

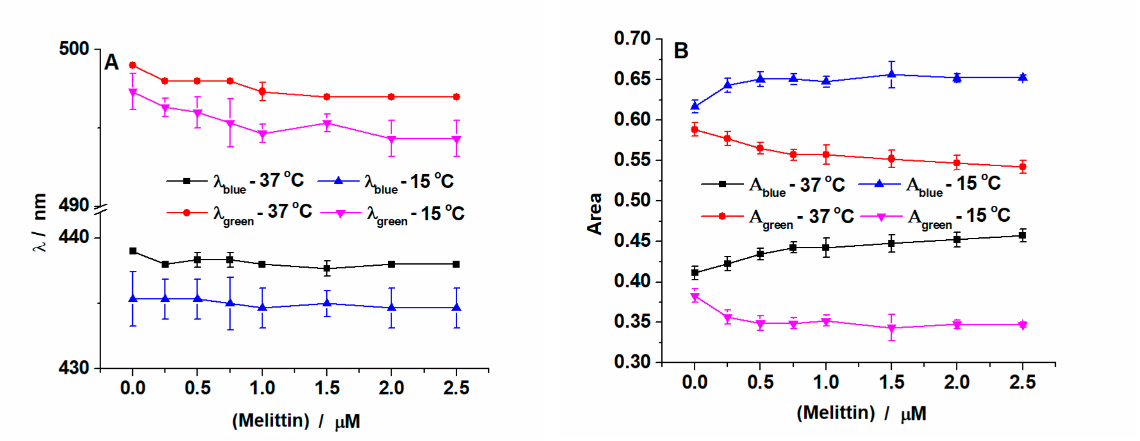

Figure S2. Variation of peak position (**A**) and area of the peak (**B**) for DOPC/Chol LUVs recorded at 15 °C and 37 °C in the presence of increasing concentrations of Melittin.

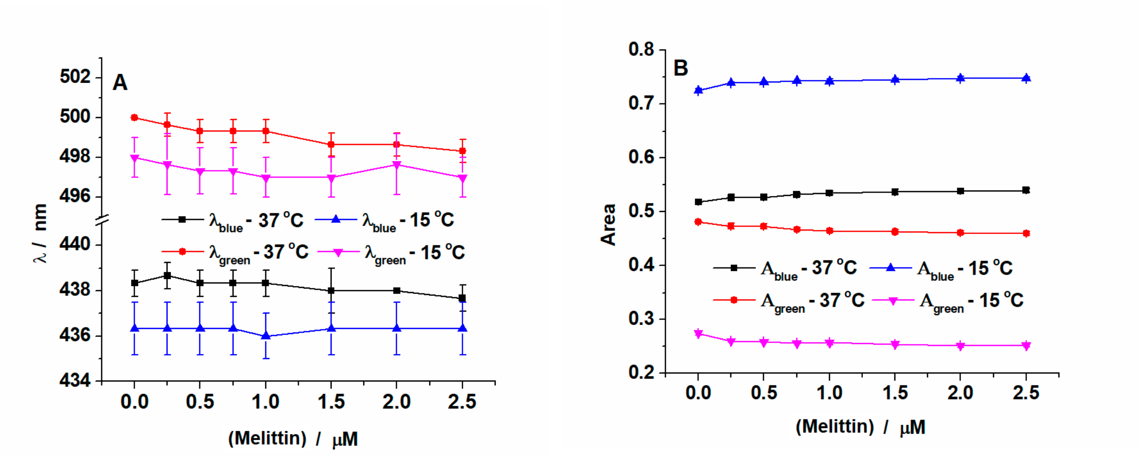

Figure S3. Variation of peak position (**A**) and area of the peak (**B**) for DOPC/Chol MLVs recorded at 15 °C and 37 °C in the presence of increasing concentrations of Melittin.

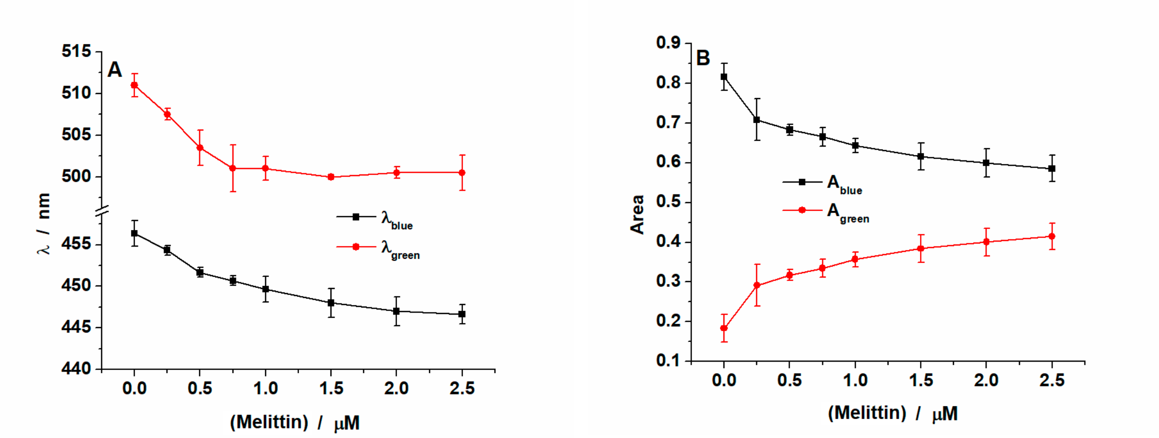

Figure S4. Variation of peak position (**A**) and area of the peak (**B**) for L929 recorded at 37 °C in the presence of increasing concentrations of Melittin.

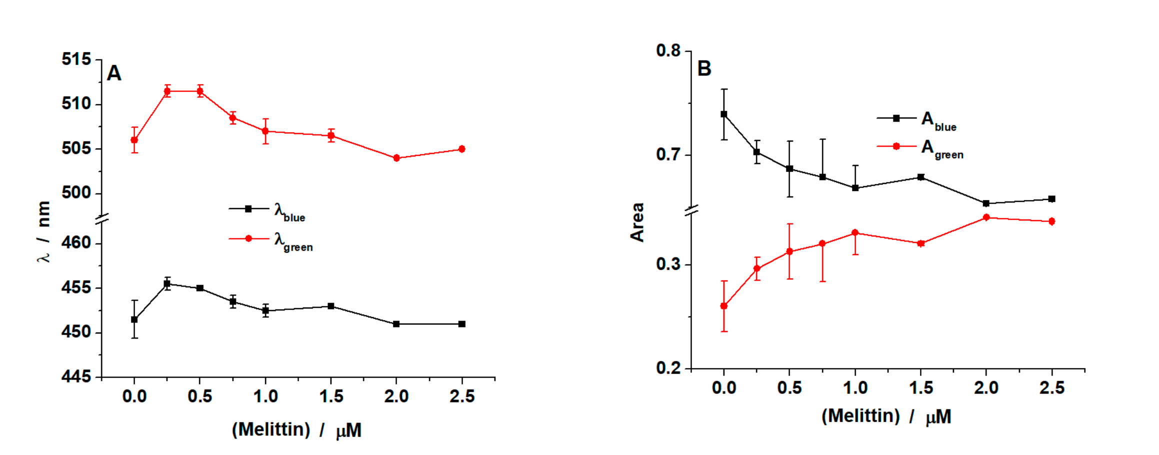

Figure S5. Variation of peak position (**A**) and area of the peak (**B**) for HT-29 recorded at 37 °C in the presence of increasing concentrations of Melittin.

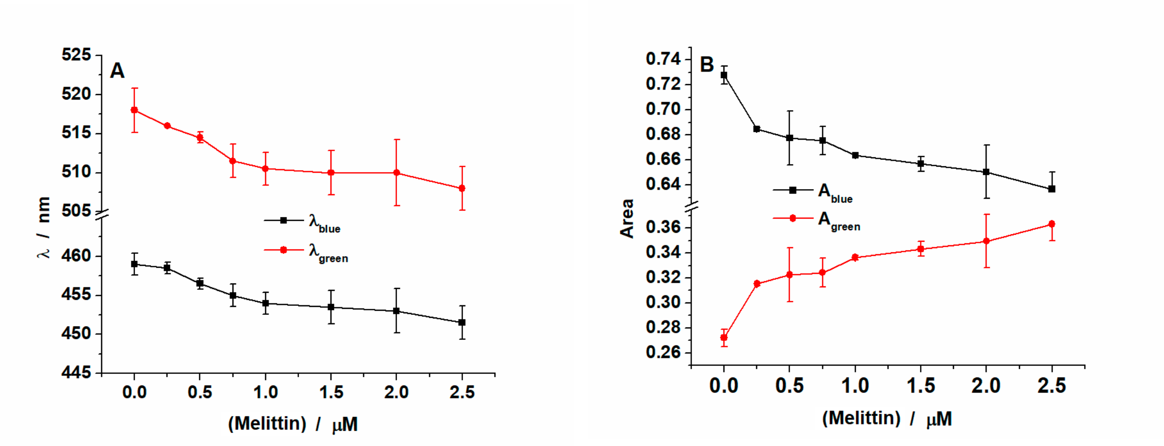

Figure S6. Variation of peak position (**A**) and area of the peak (**B**) for HepG2 recorded at 37 °C in the presence of increasing concentrations of Melittin.

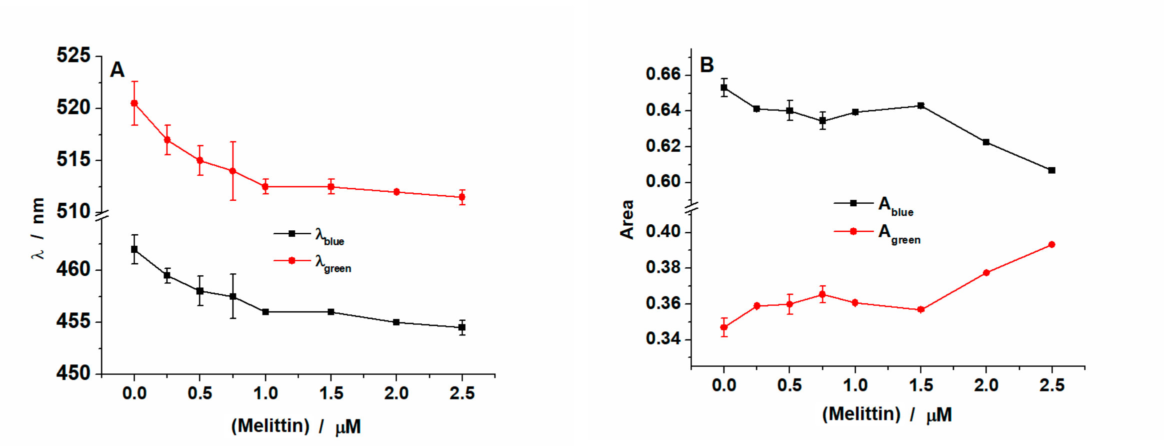

Figure S7. Variation of peak position (**A**) and area of the peak (**B**) for MG-63 recorded at 37 °C in the presence of increasing concentrations of Melittin.
